# Detection of serum antibodies to hepatitis E virus in domestic pigs in Italy using a recombinant swine HEV capsid protein

**DOI:** 10.1186/1746-6148-10-133

**Published:** 2014-06-16

**Authors:** Eleonora Ponterio, Ilaria Di Bartolo, Ginevra Orrù, Manuel Liciardi, Fabio Ostanello, Franco Maria Ruggeri

**Affiliations:** 1 Department of Veterinary Public Health and Food Safety, Istituto Superiore di Sanità, Viale Regina Elena 299, 00161 Rome, Italy; 2 S.C. Diagnostica territoriale di Cagliari, Istituto Zooprofilattico Sperimentale della Sardegna, Via dell’Acquedotto Romano - Z.I, 09030 Elmas (CA), Italy; 3 Department of Veterinary Medical Sciences, University of Bologna, Via Tolara di Sopra 50, 40064 Ozzano Emilia (BO), Italy

**Keywords:** Hepatitis E virus, Swine, ORF2, ELISA, Western blotting

## Abstract

**Background:**

The hepatitis E virus (HEV) has been detected in both humans and animals, particularly pigs, worldwide. Several evidences, including human infection following consumption of raw contaminated meat, suggest a zoonotic transmission of HEV. In Italy, large circulation of genotype 3 HEV has been reported in swine, and recent studies have confirmed the involvement of this genotype in autochthonous human cases.

**Result:**

In this study 111 sera collected from healthy pigs in two Italian regions were tested for anti-HEV IgG antibodies. For specific HEV antibody detection in swine, we developed ELISA and Western blotting methods, using a truncated capsid (ORF2) protein lacking the first 111 amino acids of a swine HEV genotype 3 strain. The ORF2-based ELISA revealed anti-HEV antibodies in 104 out of 111 pigs compared with 102 detected with a commercial ELISA kit. A lower number of sera reacted with the recombinant ORF2 protein in a Western blotting format (81/111). Using a Latent class analysis (LCA), the estimated sensitivities for ELISA-ORF2 and ELISA-kit tests were 0.961 and 0.936, respectively, whereas specificities were 0.599 and 0.475. The estimated sensitivity of Western blotting was 0.775, and the specificity was 0.944.

**Conclusions:**

The overall results confirm the high prevalence of HEV seropositive healthy pigs in Italy. Through comparisons with a commercial ELISA test, the swine genotype 3 HEV antigen produced in this study was proven suitable to detect anti-HEV antibodies in pig sera by both ELISA and Western Blotting.

## Background

Hepatitis E virus (HEV) has long been recognized as an endemic pathogen in developing countries, involved in large waterborne outbreaks. An increasing number of autochthonous cases of hepatitis E have been recently reported also in industrialized areas [1-4].

HEV is a small non enveloped RNA virus, belonging to the *Hepeviridae*[5]. The genome is a single-stranded RNA of approximately 7.3 kb, containing three open reading frames (ORFs). ORF1 encodes non-structural proteins, ORF2 the viral capsid protein, and ORF3 a cytoskeleton-associated phosphoprotein [6]. Mammalian HEV strains have been classified into four genotypes designated genotype 1 through 4 [7]. More recently, new and genetically distant viral strains have been detected in rats [8], ferrets [9], foxes [10] and bats [11], but the current HEV classification has not been modified yet. Up to date, despite a further classification of genotypes in subtypes, a single serotype has been confirmed [7].

The first HEV strains infecting animals were identified in swine [12], and later in humans [13], and were classified as genotype 3. This HEV genotype is now recognized as the most common one in both humans and swine in industrialized countries [2,3,14-16]. Several evidences support the zoonotic transmission of HEV from domestic pigs, wild boar, and deer. Furthermore, the strict correlation between animal and human strains from the same geographical areas and the numerous reports of hepatitis E cases in humans correlated with consumption of undercooked or raw meat from deer, wild boar and pig contaminated with HEV support the zoonotic transmission [4,17-20].

Usually HEV viremia and shedding have short duration in man [21-23], whereas HEV-specific serum IgG are detectable for years. The same is thought to occur in animals, and determination of HEV-specific serum antibodies can help assess the extent of past exposure to HEV in both individual herds and/or the overall animal population in a country [22]. Serological HEV studies on swine are normally conducted using commercial kits based on human HEV antigens, but use of swine virus antigens was proposed to increase testing sensitivity [24,25].

Several studies have been conducted in swine yielding different HEV seroprevalence rates, but none of the tests used was fully validated due to the absence of proper “gold standards” [24-26].

In this study, we implemented a reliable ELISA test for detection of anti-HEV antibodies in swine sera, using a genotype 3 swine HEV capsid protein expressed by a recombinant baculovirus in insect cells as coating antigen. We assessed the in house ELISA by a Latent class analysis (LCA), which permits test validation in the absence of gold standards [27]. The test was employed to evaluate the presence of HEV antibodies in 111 swine sera collected from different farms (No. 65) and a slaughterhouse (No. 46) in Italy. Results obtained with the ELISA based on recombinant swine HEV genotype 3 rΔ111ORF2 ORF2 capsid protein (ELISA-ORF2) were compared with a Western blotting (WB) test using the same antigen and with a commercial ELISA kit.

## Results

### Expression of swine HEV genotype 3 rΔ111ORF2 capsid protein in Sf9 cells

**Figure 1:**
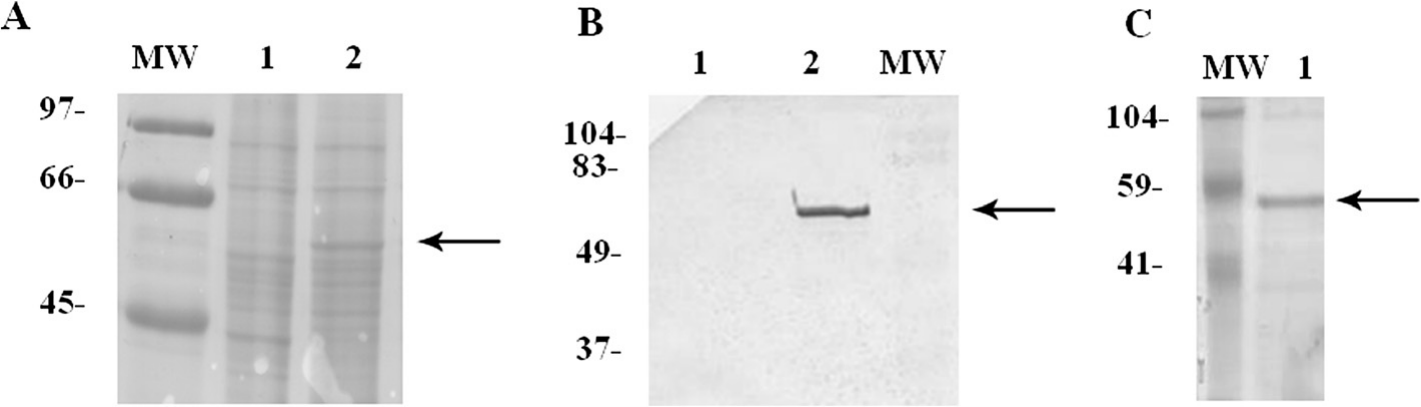
**Electrophoretic analysis of expressed HEV rΔ111ORF2 protein.** SDS-PAGE **(panel A)** and Western blotting **(panel B)** of crude protein extracts from mock infected Sf9 cells (lane 1) and cells infected with BacHEVΔ111ORF2 (lane 2). After electrophoresis, gels were stained with Coomassie Blue for direct protein visualization, or blotted onto NC paper for protein Immunostaining using an anti-HEV swine immune serum (see Methods). The 55 kDa HEV rΔ111ORF2 protein band is indicated by an arrow. **Panel C** shows the rΔ111ORF2 protein separated by PAGE and stained with Coomassie Blue, after purification using a Macro-Prep Hydroxyapatite column. MW: molecular weight markers.

Sf9 cells infected with the baculovirus BacHEVΔ111ORF2 expressed large amounts of the recombinant rΔ111ORF2 protein cloned from a genotype 3 HEV strain, as confirmed by SDS-PAGE (Figure 1A) and Western blotting (Figure 1B). The rΔ111ORF2 protein was recognized by an anti-HEV hyperimmune swine serum (kindly provided by Nicole Pavio, ANSES-ENVA-INRA, Maisons-Alfort, France) [25], but not by an SPF swine serum (not shown). The rΔ111ORF2 protein purified from Sf9 lysates showed a size of 55 kDa, corresponding to the capsid protein lacking the first 111 amino acids.Electron microscopic examination of ultracentrifuged infected cell lysates (not shown) revealed that the rΔ111ORF2 protein did not self-assemble into virus-like particles (VLP). For this reason, rΔ111ORF2 was concentrated and purified by ion-exchange chromatography (see Methods) (Figure 1C). Despite the lack of VLP formation, concentrated rΔ111ORF2 elicited a specific immune response in Balb/c mice, which showed serum antibody titers of approximately 1:1000 by both ELISA-ORF2 and Western blotting (not shown). These mouse sera were used as additional positive controls throughout the study.

### Seroprevalence of swine HEV antibodies

One hundred and four out of 111 (93.7%) swine sera were positive by the ELISA-ORF2 test, whereas 102 sera were found to be positive by the commercial ELISA (91.9%) (Table 1). Forty of the 46 pig sera taken at slaughterhouse had been found positive previously [16] by the commercial ELISA kit, and were again confirmed positive by the ELISA-ORF2 in this study despite long storage at −20°C.

**Table 1:** Detection of anti-HEV antibody in swine sera by ELISA-ORF2, ELISA-kit, and Western blotting assays

ELISA -ORF2 (%)*	**ELISA-kit (%)**	**Western blotting (%)**	**S/CO ELISA-ORF2**	**p**	**S/CO ELISA-kit**	**p**	**Observed frequency**	**Estimated frequency**	Probability of infection**
+	+	+	5.40	<0.01	5.15	<0.05	75	71	1
+	+	-	3.79		4.61		23	23	0.89
+	-	+	4.69	<0.05	1.51	<0.05	4	5	0.98
+	-	-	2.30		1.31		2	3	0.56
-	+	+	0.99	<0.05	2.10	<0.05	1	3	0.93
-	+	-	0.73		3.69		3	3	0.33
-	-	+	0.81	<0.05	1.80	<0.05	1	0	0.61
-	-	-	0.63		1.75		2	2	0.05
104 (93.7)	102 (91.9)	81 (73.0)					111		

*positive/total tested.

**estimated by the model.

**Figure 2:**
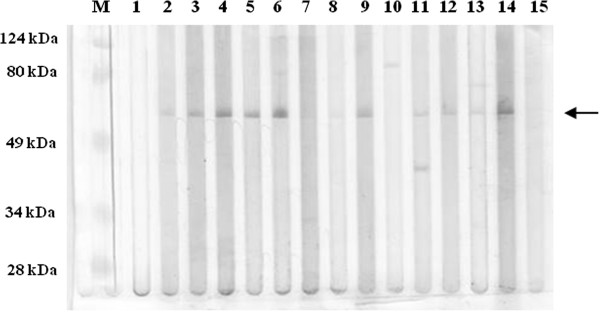
**Western blotting of the HEV r**Δ**111ORF2 capsid protein, stained with swine sera from this study (lanes 1 – 13).** Positive sera recognized a single protein band of 55 kDa (arrow). Lane 14: swine positive control serum; lane 15: SPF swine negative control serum. MW: molecular weight markers.

All swine sera were also analyzed by Western blotting using the rΔ111ORF2 protein. Eighty-one sera (73%) reacted with the rΔ111ORF2 protein specifically (part of the 81 sera are shown in Figure 2), as did the swine positive control serum. Seventy-five Western blotting positive sera were also positive by both ELISA tests, whereas four sera tested positive only at ELISA-ORF2, one at ELISA-kit, and one resulted negative at both assays (Table 1). Overall, slaughtering animals showed a seroprevalence of 86.9% (40 seropositive/46 tested), whereas all 65 farmed swine were seropositive (seroprevalence 100%).

The mean S/CO ratios between the OD values obtained for swine serum samples and the Cut-Off for each of the ELISA tests were also calculated grouping samples according to the positivity by one or both ELISA tests and WB (Table 1). Although it was overall higher for ELISA- and WB-positive samples, a statistically significant difference (p < 0.01) of the S/CO ratio was shown for the ELISA-ORF2 test among samples that were positive by all three assays.

### Validity of the ELISA-ORF2 diagnostic test

The expected and observed frequencies of HEV-positive swine sera by diagnostic test, and the probability of belonging to latent class HEV infection predicted by this model are reported in Table 1. The estimated mean sensitivities for ELISA-ORF2 and ELISA-kit tests were 0.961 (95% CI: 0.911-0.992) and 0.936 (95% CI: 0.880-0.976), respectively. The estimated mean specificities of ELISA-ORF2 and ELISA-kit tests were 0.599 (95% CI: 0.173-0.997) and 0.475 (95% CI: 0.112-0.918), respectively. The estimated sensitivity of Western blotting was 0.775 (95%CI: 0.676-0.892), and the specificity was 0.944 (95% CI: 0.886-0.979). The mean estimate of the posterior probability distribution of the unbiased proportion of seropositive swine was 0.921 (95% CI: 0.797-0.985). Credibility intervals (CI) were relatively large because of the greater uncertainty related to non-informative priors. However, the sensitivity was similar for both ELISA tests.

## Discussion

Highly sensitive molecular techniques for viral genome identification have helped increase the detection rate of HEV in swine, but the short duration of virus shedding in feces represents a major limitation when investigating the prevalence of HEV infection in farms. Serological diagnosis by immunoassays can represent a valid screening method, because HEV-specific antibodies remain detectable much longer than viral RNA. Although several HEV genotypes have been described, only one viral serotype is acknowledged to date. Tests developed for screening human serum antibodies have consequently been adapted to investigate also the swine by replacing the secondary antibodies with an anti-pig Ig serum for successful detection of specific swine antibody [28,29]. Despite the cross-reaction between genotypes, immunoassays based on swine HEV antigens have been recently suggested to provide higher sensitivity for swine serum antibody detection than those developed on human HEV strains [25,28].

The expression of a N-terminal truncated form of the ORF2 capsid protein lacking the first 111 amino acids was previously shown to favor its self-assembly in VLPs for both a genotype 1 HEV strain [30] and a rat HEV [31], suggesting that post translational processing is required for proper protein refolding [30]. Other authors [25] have reported VLP production also from swine genotype 3 ORF2, using a similar approach. Conversely, the truncated form of the swine genotype 3 capsid protein expressed in this study did not self-assemble into virus-like particles, despite being expressed in large amount. The reasons for the failure in generating VLPs in this study are not clear, and might involve different assembly or post-translational modifications of the capsid protein between different genotypes or strains of human and/or swine HEV. In fact, the capsid proteins of human genotype 1 [30] and swine genotype 3 (this study) HEV shared 94.5% amino acid identity.

Nonetheless, the unassembled swine HEV protein was recognized both efficiently and specifically by a reference swine serum raised against the naïve virus [25], and was highly immunogenic in mice, suggesting some extent of folding and conservation of its antigenic structure. In a previous study, a recombinant genotype 1 HEV peptide (HEV 239; Hecolin; Xiamen Innovax Biotech, Xiamen, China) was found to occur in solution as large aggregates rather than true VLPs [32], but it was highly immunogenic. In this form, HEV 239 has been used as an efficacious recombinant hepatitis E vaccine for human use [33].

The suitability of the rΔ111ORF2 protein for testing HEV antibody in swine sera using ELISA was confirmed by comparing by LCA the in-house method with a commercial ELISA-kit test, produced for human use and present in the market since several years. Sensitivity of the commercial assay is expected to be high, since antigens of different species and strains are known to cross-react largely, in line with the knowledge of a single serotype among all HEV genotypes [23,34,35].

By the LC model adopted, ELISA-ORF2 and ELISA-kit presented sensitivities of 0.961 and 0.936, and specificities of 0.599 and 0.475, respectively. The low specificity value for both ELISAs is due to the assumption that the immune-stained protein band in the Western blotting assay is 100% specific, but does not consider that Western blotting can yield false-negative results. Therefore, the ORF2 band absence in Western blotting using sera that reacted in ELISA does not imply a corresponding false-positive result of ELISA. The ELISA-kit flyers indicate that specificity varies between 92.7 and 100% with human sera [36-38], even if some tests can cross-react with Epstein-Barr virus (EBV) and cytomegalovirus (CMV) antibodies, determining false positive results [39]. Therefore, it is hard to believe that the ELISA-kit would perform very poorly in terms of specificity only when adapted to detection of antibodies from a different species, such as the swine.

In addition, the normalized ratios between the OD values determined for test sera and the Cut-Off ODs for the ELISA tests used (S/CO value) were higher for swine sera that also proved positive at WB analysis. Although a statistically different S/CO was confirmed only in the case of ELISA-ORF2 for the more numerous group of samples that were positive at all three tests, these results overall suggest that positivity at WB is correlated with higher ELISA OD values, which may imply a higher serum antibody titer.

In this study, we have determined a mean anti-HEV seroprevalence of up to 93.7% by ELISA. No differences were observed depending on the geographical origin of farmed swine, whereas a lower seroprevalence of anti-HEV IgG in pigs from slaughterhouse was observed. This might be due to a decline of infection rate and/or immunity among the older animals in this stage of pork production [23]. In fact, most of domestic pigs get infected at 2–3 months of age [40], and even if animals are in contact with the virus throughout their life span they could be seronegative at slaughtering age [41]. Furthermore, 25 of 40 (62.5%) HEV-seropositive pigs collected at slaughterhouse were found to be HEV infected by HEV genome testing in bile, liver and/or feces [16]. This result confirms that swine remains susceptible to HEV infection at any age, even at slaughter, that in Italy normally involves animals of 9 months of age and more.

## Conclusions

Overall the results presented in this paper confirm that the swine HEV rΔ111ORF2 may be suitably applied to large seroprevalence studies in pig herds using an ELISA format. We found the in house assay to be at least as sensitive as the commercial ELISA kit including a human genotype viral antigen. These data support further that the human and swine HEV strains belonging to different genotypes are highly cross-reactive, if not even identical, in their antigenic determinants. Finally, the use of over one-hundred swine sera for diagnostic assays comparisons in this study permitted to determine a mean anti-HEV seroprevalence of up to 93.7% among pigs which are part of the Italian food chain, confirming the high HEV circulation among Italian farmed pigs previously reported in Italy and other countries [16,18,24,25,28].

## Methods

### Generation of recombinant baculovirus and expression of HEV capsid protein

Total RNA was extracted from the HEV-positive pig bile sample SwHEV/BO85/06 collected in Northern Italy, using the Qiamp Viral RNA Extraction kit (Qiagen). The cDNA corresponding to HEV ORF2 was obtained using oligo(dT)_20_ primer and SuperScript^™^III reverse transcriptase (First-Strand SuperScript^™^III Synthesis System, Life Technologies), following the manufacturer’s instructions. Amplification of an ORF2 fragment lacking the first 333 nucleotides was performed by PCR with primers designed on an Italian swine HEV ORF2 available in [GenBank NCBI: GU117636], denominated F∆111HEV (5′-GC*TCTAGA*GC**ATG**GCCGTATCACCGGCTCCCGATACA GCC-3′), flanked by *Xba*I restriction site (italics), and RHEV (5′-GAC*TCGAGA***TTA**AGACTC CCGGGTTTTACC-3′), flanked by *Xho*I site (italics) and annealing in the ORF2 3′-terminal portion (stop codon indicated in bold). A methionine codon (in bold in F∆111HEV primer) was introduced into the forward primer F∆111HEV between *Xba*I cleavage site and the alanine codon at position 112. The 1652 bp DNA fragment obtained, flanked by *Xba*I (italics) and *Xho*I sites, was ligated into the pFastBac™1 (Life Technologies) donor vector. The construct obtained, which was named pFast::HEVΔ111ORF2, was transformed in the *E. coli* DH10Bac™ host strain containing a Baculovirus shuttle vector (bacmid) and a helper plasmid. The recombinant baculovirus DNA BacHEVΔ111ORF2 was generated by transposition in the *E. coli* DH10Bac host, and the resulting bacmid was purified and transfected into Sf9 cells using Cellfectin-II (Life Technologies) to produce infectious recombinant baculovirus. Based on comparison of ORF2 sequence with HEV reference strains available on GenBank, the identity of the HEV swine strain as genotype 3 subtype e, and its correct reading frame was confirmed [GenBank NCBI: GU556929].

### Antigen preparation and purification

Sf9 cell monolayers grown in Sf900 medium (Life Technologies) in ten T75 cm^2^ flasks were infected with BacHEVΔ111ORF2 baculovirus. When a diffuse cytopathic effect was observed, cell cultures were lysed by three cycles of freezing and thawing. The recombinant protein rΔ111ORF2 was partially purified from the supernatant by anion exchange chromatography using a Macro-Prep Hydroxyapatite column (Bio-Rad), following the manufacturer’s instructions. A 55 kDa protein corresponding to the 111-aa deletion fragment of the capsid protein was produced, and analyzed by SDS-PAGE.

### Immunization of Balb/c mice with recombinant HEV capsid protein

Animal work was conducted according to the Italian legislation enacting the EU directives (D.L. 116/92), following approval of the specific experimental protocols (mouse immunization and serum withdrawal, granted to Franco Maria Ruggeri) by the Biological and Animal Experimental Managing Service of the Istituto Superiore di Sanità and the legal authorization by the Italian Ministry of Health (Decree no. 97/2011 – B, 24th May 2011).

Three adult Balb/c mice (12 week-old) were immunized three times intraperitoneally at 3-week intervals with 15 μg of purified HEV capsid protein, in the presence of complete Freund’s adjuvant for the first inoculation, and incomplete adjuvant for the following immunizations.

A mouse inoculated with sterile saline solution was used as negative control. Fourteen days after the final boosting, sera were tested for anti-HEV antibodies by Western blotting, as described below [25]. Mice were euthanized following isoflurane anesthesia, and serum was collected from the intracardiac clot.

### Swine sera

Sixty-five sera were aliquots from a larger sample of sera previously analyzed and stored within the regional monitoring plan for African swine fever (ASF) and Classical swine fever (CSF) enforced in Sardinia, in compliance with the European Community requirements. Sera had been collected from clinically healthy pigs by staff veterinarians of the Italian Public Health System (ASL), according to Decrees No. 9, 16.05.2007, and No. 1567/decA/23, 14.07.2009, of the “Assessorato dell’Igiene e Sanità e dell’Assistenza Sociale, Regione Autonoma della Sardegna”, at five farms located in different areas in Sardinia [42]. Additional 46 sera had been collected post-mortem from the intracardiac clot of slaughtered pigs at pork slaughterhouses in Northern Italy [16]. Sera were stored at −20°C until use.

### Western blotting

Purified rΔ111ORF2 was separated by SDS-PAGE, and either stained with Coomassie Brilliant Blue R-250 (Bio-Rad), or transferred to nitrocellulose membrane (Trans-blot transfer medium, Bio-Rad). After blocking with 5% skim milk in phosphate-buffered saline (PBS), the membrane was incubated with test (diluted 1:160) or control (1:1000) sera in PBS containing 0.05% Tween-20 and 1% skim milk, for 4 hours. Three sera from Specific Pathogen Free (SPF) pigs were used as negative control, while an experimentally infected swine serum [25] and a hyperimmune anti-HEVORF2 mouse serum were used as positive controls. Membranes were then incubated with alkaline phosphatase-conjugated anti-pig IgG (1:12000; SIGMA) or anti-mouse IgG (1:3000; Bio-Rad). Bands were visualized with 1-step NBT/BCIP solution (Pierce).

### Recombinant ORF2-based ELISA procedure (ELISA-ORF2)

Polystyrene 96-well microplates (Maxisorp, Nunc) were coated with purified rΔ111ORF2 (0.01 μg/well). A control plate was coated with an Sf9 lysate containing an irrelevant protein (bovine norovirus capsid) [43]. After 18 hours at 4°C, wells were washed 3 times with PBS containing 0.05% Tween-20 (PBS-T), and blocked with 5% skim milk in PBS at 37°C for 2 hours. After washings, plates were incubated with test sera diluted 1:20 in PBS containing 0.05% Tween-20 and 2% skim milk (PBS-T-milk), at 37°C for 90 min, in triplicate. Swine positive and control sera were used in all tests.

After washing with PBS-T, anti-pig (1:12000) and anti-mouse (1:3000) conjugates were added. Following 1 hour incubation at 37°C and washings, the reaction was developed with p-nitrophenol phosphate (SIGMA) in 10 mM diethanolamine, pH 10, for 1 hour at 37°C. Optical densities were measured at a wavelength of 405 nm (OD_405_). The Cut-Off value (COV) for the ELISA-ORF2 was established from internal controls for each test, using the commercial ELISA test formula: [mean OD of negative controls + 0.1]. Throughout this study, this value was 0.18.

### Commercial ELISA test (ELISA-kit)

Swine sera (diluted 1:20) were also tested by the BioChain kit (http://www.biochain.com, USA), commercially available for anti-HEV IgG detection in human sera, with the following modifications: the secondary antibody was replaced with a horseradish peroxidase-labeled anti-swine IgG antibody (SIGMA), diluted 1:10000. Test sera were considered as positive if OD_450_ ≥ COV, as indicated by the kit manufacturer. Positive and negative swine control sera (see above) were included in all assays.

All reporting adheres to the NC3Rs ARRIVE guidelines (Animal Research: Reporting In Vivo Experiments) (Additional file 1).

### Statistical analysis

A Bayesian approach was used to obtain estimates for the test accuracies of the three tests. The data were processed with Bayes Latent Class Models software (http://www.medicine.mcgill.ca/epidemiology/Joseph/Bayesian-Software-Diagnostic-Testing.html). Parameter estimates were based on analytical summaries of 10,000 iterations of the Gibbs sampler with a burn-in phase of 1000 iterations.

Sensitivity and specificity were estimated by the classic validation method as well as by Latent-class analysis modeling. In Latent class analysis (LCA), we started by fitting the basic two latent class model.

Beta distributions Be (a, b) were used as priors for the parameters of interest (sensitivities, and specificities). The same Beta prior distributions [25] were used for the sensitivities of both ELISA tests: <0.6; mode = 0.9 corresponding to Be (8.30, 1.81) priors. For both ELISA tests a uniform prior distribution for specificity Be (0, 1) was assumed.

Swine sera testing by HEV WB were considered 100% specific. The Beta prior distribution for WB specificity had mode = 0.9 and a 5^th^ percentile of 0.9. For WB tests we assumed a uniform prior distribution for sensitivity Be (0, 1).

Sensitivity and specificity of tests were reported with an approximate 95% confidence interval (CI), as estimated by the model.

## Competing interests

All authors read and approved the final manuscript, and agreed with the conclusions of the work. The authors declare that they have no competing interests.

## Authors’ contributions

EP participated to cloning and to production of protein, conducted serological testing, and contributed to data analysis and drafted the first version of the manuscript. IDB participated to cloning, supervised laboratory testing, and contributed to data analysis and participated to final draft preparation. GO and ML organized and performed animal sampling and sera collection. FO performed statistical analysis, and participated to final draft preparation. FMR supervised the activities, and reviewed the final manuscript. All authors read and approved the final manuscript.

## Supplementary Material

Additional file 1 NC3Rs ARRIVE Guidelines Checklist. Click here for file
